# Early postoperative hypoalbuminaemia is associated with pleural effusion after donor hepatectomy: A propensity score analysis of 2316 donors

**DOI:** 10.1038/s41598-019-39126-0

**Published:** 2019-02-26

**Authors:** Hye-Won Jeong, Jung-Won Kim, Won-Jung Shin, Seon-Ok Kim, Young-Jin Moon, Hye-Mee Kwon, Kyeo-Woon Jung, In-Gu Jun, Jun-Gol Song, Gyu-Sam Hwang

**Affiliations:** 1 Department of Anaesthesiology and Pain Medicine, International St. Mary’s Hospital, Catholic Kwandong University College of Medicine, Incheon, Korea; 2 Department of Anaesthesiology and Pain Medicine, CHA Bundang Medical Center, CHA University School of Medicine, Seongnam, Korea; 3 Department of Anaesthesiology and Pain Medicine, Laboratory for Cardiovascular Dynamics, Asan Medical Center, University of Ulsan College of Medicine, Seoul, Korea; 4 Department of Clinical Epidemiology and Biostatistics, Asan Medical Center, University of Ulsan College of Medicine, Seoul, Korea

## Abstract

Pleural effusion and hypoalbuminaemia frequently occur after hepatectomy. Despite the emphasis on the safety of donors, little is known about the impact of postoperative albumin level on pleural effusion in liver donors. We retrospectively assessed 2316 consecutive liver donors from 2004 to 2014. The analysis of donors from 2004 to 2012 showed that postoperative pleural effusion occurred in 47.4% (970/2046), and serum albumin levels decreased until postoperative day 2 (POD2) and increased thereafter. In multivariable analysis, the lowest albumin level within POD2 (POD2ALB) was inversely associated with pleural effusion (OR 0.28, 95% CI 0.20–0.38; *P* < 0.001). POD2ALB ≤3.0 g/dL, the cutoff value at the 75th percentile, was associated with increased incidence of pleural effusion after propensity score (PS) matching (431 pairs; OR 1.69, 95% CI 1.30–2.21; *P* < 0.001). When we further analysed data from 2010 to 2014, intraoperative albumin infusion was associated with higher POD2ALB (*P* < 0.001) and lower incidence of pleural effusion (*P* = 0.024), compared with synthetic colloid infusion after PS matching (193 pairs). In conclusion, our data showed that POD2ALB is inversely associated with pleural effusion, and that intraoperative albumin infusion is associated with a lower incidence of pleural effusion when compared to synthetic colloid infusion in liver donors.

## Introduction

Living donor liver transplantation (LDLT) has become a standard therapeutic option for patients with end-stage liver disease. It has partially relieved the intractable shortage of deceased donor grafts worldwide^1,2^, with recipient survival rates comparable to those of cadaveric donor liver transplantation^3,4^. However, the safety of living liver donors remains a major concern, requiring a delicate balance between the potential risk to the donor and the benefit to the recipient. According to a recent survey, the incidences of donor morbidity and mortality were 24% and 0.2%, respectively^5^.

Respiratory complications are common after living donor hepatectomy, and pleural effusion is one of the most common complications, occurring in 20.4–41.0% of all donors^6–8^. Although it is mostly asymptomatic and spontaneously absorbed, symptomatic pleural effusion is responsible for hypoxaemia, dyspnoea, and pyrexia requiring thoracentesis or thoracotomy in 3.9–5.0% of all liver donors^6–8^. Considering the significant risk to healthy donors, careful perioperative care with a rational approach is essential for minimizing even mild complications^9^.

Postoperative hypoalbuminaemia is frequently seen in donors after hepatectomy for LDLT^10,11^, owing to dilution secondary to fluid administration, altered redistribution and vascular permeability, increased catabolism, and reduced hepatic synthesis^12–14^. Studies have shown that postoperative hypoalbuminaemia is associated with poor outcomes in patients undergoing major surgery^15,16^. Moreover, hypoalbuminaemia is considered to be a contributing factor in the occurrence of pleural effusion^17,18^. Specifically, the ability of albumin to maintain 75–80% of the plasma colloid osmotic pressure (COP), to influence vascular membrane permeability, and to act as an antioxidant can reduce pulmonary effusion and improve pulmonary oxygenation function^19,20^. Although several previous reports have demonstrated that hypoalbuminaemia is related to pleural effusion in various situations^18,21^, the relationship of transient postoperative hypoalbuminaemia with the occurrence of pleural effusion in healthy living donors has not been well elucidated. Therefore, we sought to investigate the association of early postoperative albumin level with the development of pleural effusion after donor hepatectomy for LDLT. We also assessed the effect of intraoperative use of albumin solution instead of synthetic colloid on postoperative albumin levels and the incidence of pleural effusion.

## Results

**Figure 1:**
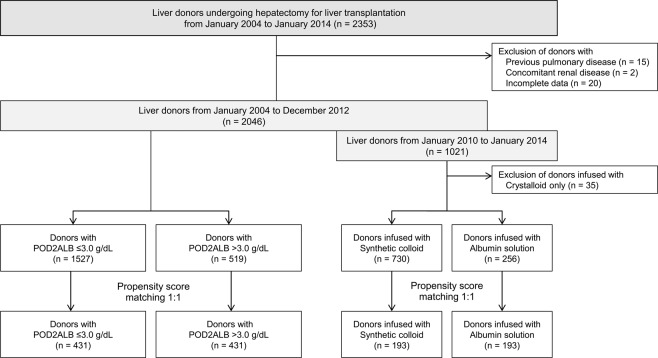
Flowchart of the study population. Abbreviation: POD2ALB, lowest albumin level within postoperative day 2.

A total of 2353 living liver donors who underwent donor hepatectomy for LDLT between January 2004 and January 2014 were enrolled. Of these, 37 donors were excluded from the analysis, including 15 who had previous pulmonary disease, 2 who had renal disease, and 20 with incomplete data (Fig. 1). Synthetic colloids were substituted by albumin solution in LDLT donors from January 2013 at our institution due to the potential risk of acute kidney injury (AKI) associated with their use^22,23^. Therefore, we performed analyses for two periods: 2004–2012 (synthetic colloid use) and 2010–2014 (synthetic colloid use from 2010 to 2012 vs. albumin use from 2013 to 2014), as described in Fig. 1.

**Figure 2:**
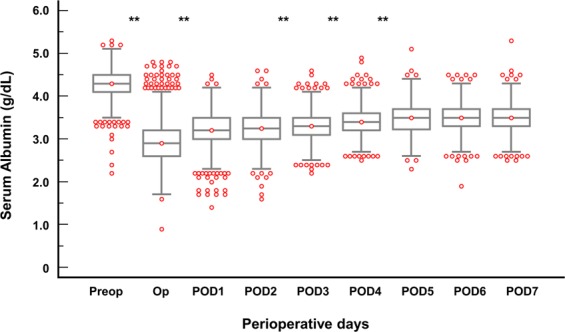
Sequential changes in serum albumin levels in donors undergoing donor hepatectomy. *P*-value is the result of comparing the adjacent groups. **P < 0.001. Abbreviations: Preop, preoperative day; Op, operation day; POD, postoperative day.

We first analysed the association between postoperative albumin level and the development of pleural effusion in the donors who underwent hepatectomy from 2004 to 2012 (n = 2046) (Tables 1–4 and Fig. 2). Serum albumin level was measured from preoperative day 1 to postoperative day (POD) 7 according to our routine institutional protocol for LT donors. We found that the increase of postoperative albumin level stagnated until POD 2 and then continued to significantly increase thereafter (Fig. 2). This examination of albumin levels and the occurrence of pleural effusion after hepatectomy revealed that the lowest albumin level within POD 2 (POD2ALB) is an early and clinically relevant parameter to assess the association with the development of pleural effusion. Therefore, donors were classified into two groups according to POD2ALB (≤3.0 g/dL [n = 1527] and >3.0 g/dL [n = 519]; the cut-off value of 3.0 was defined by the 75^th^ percentile of POD2ALB). Propensity score (PS) matching was used to adjust the baseline differences (n = 431 pairs) (Table 3).

**Table 1:** Baseline characteristics and intraoperative data of liver donors from 2004–2012.

Variables	Total (n = 2046)	No pleural effusion (n = 1076)	Pleural effusion (n = 970)	*P*-value
**Preoperative variables**				
Age (years)	27 (22–33)	27 (21–33)	27 (22–33)	0.699
Sex, male	1412 (69.0)	742 (69.0)	670 (69.1)	0.994
Body mass index (kg/m^2^)	22.8 ± 2.8	22.7 ± 2.8	23.0 ± 2.7	0.028
Haemoglobin (g/dL)	14.5 (12.8–15.5)	14.3 (12.6–15.4)	14.6 (13.0–15.5)	<0.001
Platelet count (×10^9^/L)	238.0 (212.0–264.0)	238.0 (214.0–260.0)	238.0 (209.0–269.0)	0.684
Prothrombin time (INR)	1.00 ± 0.06	1.01 ± 0.06	1.00 ± 0.07	0.034
Creatinine (mg/dL)	0.8 ± 0.2	0.8 ± 0.2	0.8 ± 0.2	0.184
Albumin (g/dL)	4.3 (4.0–4.5)	4.3 (4.1–4.5)	4.3 (4.0–4.5)	0.712
Total bilirubin (mg/dL)	0.8 (0.7–1.0)	0.8 (0.7–1.1)	0.8 (0.7–1.0)	0.641
Aspartate aminotransferase (IU/L)	19.0 (16.0–22.0)	19.0 (16.0–22.0)	19.0 (17.0–22.0)	0.104
Alanine transaminase (IU/L)	16.0 (12.0–21.0)	15.0 (12.0–21.0)	16.0 (12.0–22.0)	0.068
**Intraoperative variables**				
Anaesthetic time (min)	495.0 (435.0–570.0)	500.0 (433.0–580.0)	491.0 (440.0–555.0)	0.073
Right hepatectomy	1851 (90.5)	949 (88.2)	902 (93.0)	<0.001
Crystalloid (L)	3.6 (2.9–4.6)	3.6 (2.8–4.7)	3.7 (3.0–4.5)	0.785
Synthetic colloid (mL)	500.0 (500.0–500.0)	500.0 (500.0–500.0)	500.0 (500.0–500.0)	0.103
Urine output (mL)	707.5 (480.0–1015.0)	730.0 (497.5–1067.5)	687.5 (470.0–980.0)	0.006
Diuretics use	976 (47.7)	534 (49.6)	442 (45.6)	0.073
Ephedrine use	376 (18.4)	168 (15.6)	208 (21.4)	0.001
Transfusion	7 (0.3)	2 (0.2)	5 (0.5)	0.370

Values are expressed as mean ± standard deviation, median (interquartile range), or number of donors (%), as appropriate.

Abbreviations: INR, international normalized ratio.

**Table 2:** Univariate and multivariate analysis for predicting the occurrence of postoperative pleural effusion.

	Univariate				Multivariate			
	OR	95% CI		*P*-value	OR	95% CI		*P*-value
Age	1.003	0.992	1.014	0.581				
Sex	1.005	0.833	1.213	0.956				
Body mass index	1.036	1.004	1.069	0.028	1.053	1.018	1.089	0.003
Anaesthetic time	0.999	0.998	1.000	0.017				
Right hepatectomy	1.775	1.304	2.416	<0.001	1.521	1.095	2.114	0.012
Crystalloid (L)	0.949	0.883	1.019	0.151				
Synthetic colloid (L)	1.405	0.921	2.141	0.114				
Urine output (L)	0.774	0.637	0.941	0.010				
Diuretics use	0.850	0.714	1.011	0.066				
POD2ALB (g/dL)	0.393	0.301	0.512	<0.001	0.277	0.203	0.379	<0.001

Adjusted by all variables in Table 1.

Abbreviations: POD2ALB, lowest albumin level within postoperative day 2; OR, odds ratio; CI, confidence interval.

**Table 3:** Baseline characteristics and intraoperative data of unmatched and propensity score matched donors.

Variables	Unmatched (n = 2046)			Propensity score matched (n = 862)			
	POD2ALB ≤3.0 g/dL (n = 1527)	POD2ALB >3.0 g/dL (n = 519)	Standardized difference	POD2ALB ≤3.0 g/dL (n = 431)	POD2ALB >3.0 g/dL (n = 431)	*P*-value	Standardized difference
**Preoperative variables**							
Age (years)	28.3 ± 8.2	26.3 ± 7.2	0.285	25.9 ± 7.1	26.5 ± 7.3	0.232	0.079
Sex, male	956 (62.6)	456 (87.9)	0.773	374 (86.8)	369 (85.6)	0.596	0.036
BMI (kg/m^2^)	22.8 ± 2.8	23.1 ± 2.6	0.110	22.9 ± 2.7	22.9 ± 2.6	0.959	0.004
Haemoglobin (g/dL)	14.0 ± 1.7	14.7 ± 1.4	0.522	14.7 ± 1.5	14.6 ± 1.4	0.546	0.043
Platelet count (×10^9^/L)	240.4 ± 44.4	238.5 ± 45.4	0.041	239.4 ± 42.6	238.3 ± 44.7	0.703	0.025
PT (INR)	1.00 ± 0.07	1.01 ± 0.06	0.069	1.01 ± 0.06	1.01 ± 0.06	0.856	0.012
Creatinine (mg/dL)	0.8 ± 0.2	0.9 ± 0.1	0.383	0.9 ± 0.1	0.9 ± 0.2	0.974	0.002
Albumin (g/dL)	4.2 ± 0.3	4.4 ± 0.3	0.587	4.4 ± 0.3	4.4 ± 0.3	0.461	0.043
Total bilirubin (mg/dL)	0.9 ± 0.3	0.9 ± 0.3	0.155	0.9 ± 0.3	0.9 ± 0.3	0.485	0.045
AST (IU/L)	20.1 ± 6.7	20.2 ± 8.3	0.001	20.3 ± 4.8	20.0 ± 8.8	0.010	0.031
ALT (IU/L)	17.6 ± 9.0	18.6 ± 11.2	0.089	18.6 ± 8.8	18.2 ± 11.6	0.040	0.035
**Intraoperative variables**							
Anaesthetic time (min)	520.5 ± 98.2	465.7 ± 84.6	0.648	478.5 ± 89.4	474.9 ± 85.6	0.492	0.043
Right hepatectomy	1389 (91.0)	462 (89.0)	0.062	386 (89.6)	380 (88.2)	0.502	0.045
Crystalloid (L)	4.0 ± 1.2	3.2 ± 1.0	0.792	3.4 ± 1.0	3.4 ± 1.0	0.105	0.097
Synthetic colloid (mL)	538.8 ± 205.6	466.7 ± 198.9	0.363	484.6 ± 197.1	486.5 ± 197.2	0.853	0.009
Urine output (mL)	837.8 ± 456.1	720.3 ± 437.0	0.269	751.1 ± 411.6	733.9 ± 447.1	0.405	0.039
Diuretics use	765 (50.1)	211 (40.7)	0.192	187 (43.4)	178 (41.3)	0.532	0.043
Ephedrine use	279 (18.3)	97 (18.7)	0.011	88 (20.4)	83 (19.3)	0.678	0.030
Transfusion	6 (0.4)	1 (0.2)	0.046	1 (0.2)	1 (0.2)	1.000	<0.001

Values are expressed as mean ± standard deviation, or number of donors (%), as appropriate.

Abbreviations: POD2ALB, lowest albumin level within postoperative day 2; BMI, body mass index; PT, prothrombin time; INR, international normalized ratio; AST, aspartate aminotransferase; ALT, alanine transaminase.

**Table 4:** Predictive value of POD2ALB ≤3.0 g/dL for the occurrence of postoperative pleural effusion.

		Unadjusted				Multivariable adjusted^a^			Propensity score-matched			
		Event/n	OR	95% CI	*P*-value	OR	95% CI	*P*-value	Event/n	OR	95% CI	*P*-value
Pleural effusion	POD2ALB ≤3.0 g/dL	763/1527	1.51	1.23–1.84	<0.001	1.73	1.38–2.16	< 0.001	225/431	1.69	1.30–2.21	<0.001
	POD2ALB >3.0 g/dL	207/519	1						169/431	1		

^a^Adjusted by all variables in Table 1.

Abbreviations: POD2ALB ≤3.0 g/dL, lowest albumin level within postoperative day 2 ≤3.0 g/dL; OR, odds ratio; CI, confidence interval.

**Figure 3:**
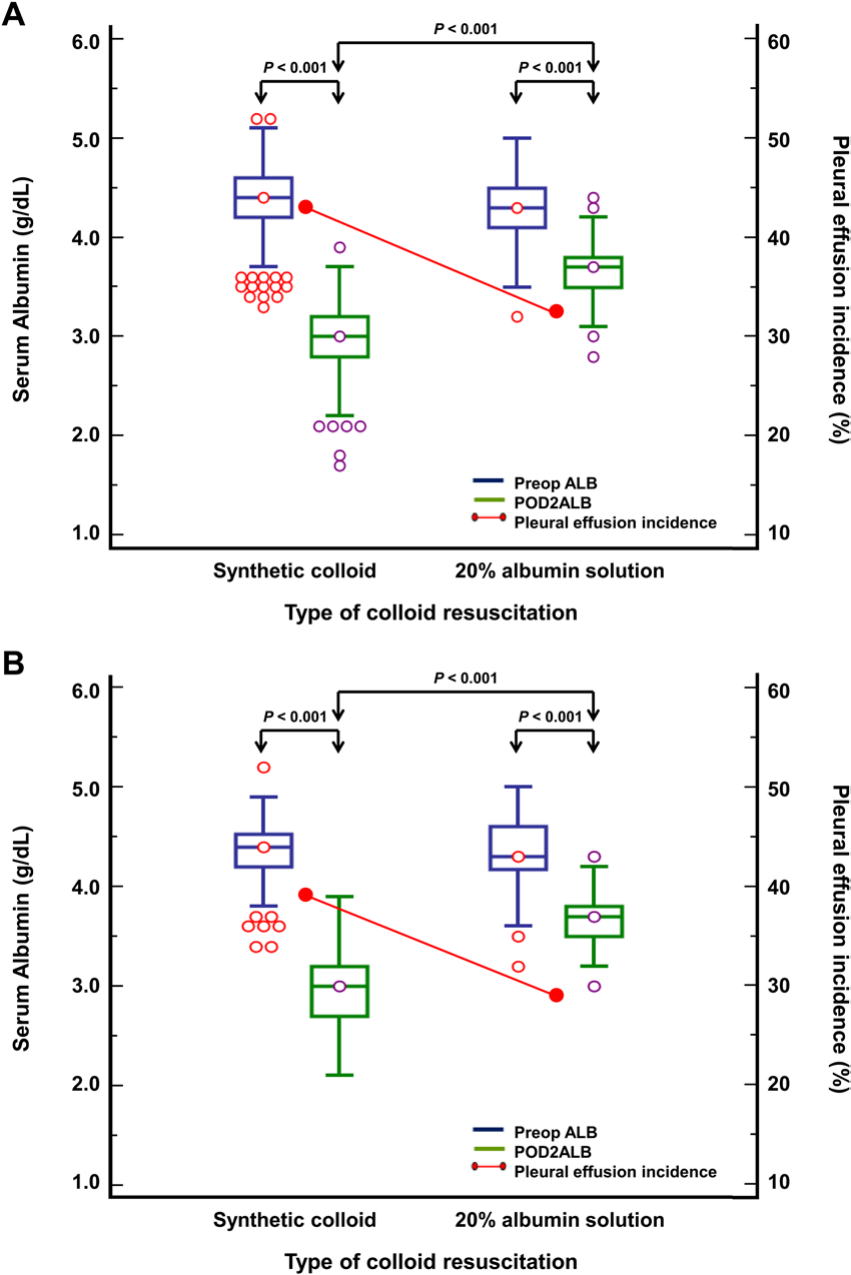
Early changes in postoperative albumin levels and the incidence of pleural effusion according to the type of colloid resuscitation (**A**. Unmatched, **B**. PS-matched). Abbreviations: PS, propensity score; Preop ALB, preoperative albumin level; POD2ALB, lowest albumin level within postoperative day 2.

We further assessed the effect of intraoperative albumin solution infusion instead of synthetic colloid on postoperative albumin levels and the incidence of pleural effusion in donors who underwent hepatectomy from 2010 to 2014 (n = 1021) (Fig. 3 and Supplementary Tables S1 and S2). Of the 1021 liver donors at our institution from 2010 to 2014, 35 who were infused with crystalloid only (neither synthetic colloid nor albumin solution for resuscitation) during donor hepatectomy were excluded (Fig. 1). A total of 730 donors infused with synthetic colloid and 256 infused with 20% albumin solution were included in the final comparison. PS matching was used to adjust the baseline differences between the groups (n = 193 pairs) (Supplementary Table S1).

Table 1 presents the baseline characteristics and intraoperative data of the included donors classified according to the occurrence of postoperative pleural effusion. Donors with postoperative pleural effusion (n = 970, 47.4%) were more likely to have undergone right hepatectomy, had more intraoperative ephedrine use, and produced less intraoperative urine than those without postoperative pleural effusion (*P * < 0.001, *P* = 0.001 and *P* = 0.006, respectively). Therapeutic thoracentesis was performed in 19 (2.0%) donors with postoperative pleural effusion. The incidence of AKI according to the Kidney Disease: Improving Global Outcomes (KDIGO) criteria was 4.2% (45/1076) in donors without pleural effusion and 4.3% (42/970) in donors with pleural effusion (*P* = 0.869).

Multiple logistic regression analysis revealed that higher body mass index (BMI) (odds ratio [OR] 1.05, 95% confidence interval [CI] 1.02–1.09; *P * = 0.003), right hepatectomy (OR 1.52, 95% CI 1.10–2.11; *P* = 0.012), and lower POD2ALB (OR 0.28, 95% CI 0.20–0.38; *P < *0.001) were associated with a higher incidence of pleural effusion (Table 2). POD2ALB ≤3.0 g/dL was significantly associated with a higher incidence of pleural effusion in both multivariable analysis (OR 1.73, 95% CI 1.38–2.16; *P* < 0.001) and PS-matched analysis (OR 1.69, 95% CI 1.30–2.21; *P* < 0.001) (Table 4). Even among the donors undergoing right hepatectomy, POD2ALB ≤3.0 g/dL was associated with a higher likelihood of postoperative pleural effusion in multivariable analysis (OR 1.54, 95% CI 1.28–1.86; *P* < 0.001).

Figure 3 presents the early changes in postoperative albumin levels and the incidence of pleural effusion among unmatched and PS-matched donors. Donors infused with albumin had significantly higher POD2ALB (Unmatched, median 3.7 [interquartile range 3.5–3.8] vs. 3.0 [2.8–3.2], *P* < 0.001 and PS-matched, 3.7 [3.5–3.8] vs. 3.0 [2.7–3.2], *P* < 0.001) and lower incidence of pleural effusion (Unmatched, 83 [32.4%] vs. 317 [43.4%], *P* = 0.002 and PS-matched, 55 [28.5%] vs. 76 [39.4%], *P* = 0.024), compared to those infused with synthetic colloid during donor hepatectomy. The use of albumin was associated with significantly reduced incidence of postoperative pleural effusion before (OR 0.63, 95% CI 0.46–0.84; *P* = 0.002) and after PS matching (OR 0.61, 95% CI 0.41–0.92; *P* = 0.018) when compared to synthetic colloid use. However, no association was observed between the incidence of AKI and the type of colloid used either before (OR 0.16, 95% CI 0.02–1.17; *P* = 0.070) or after PS matching (OR 0.16, 95% CI 0.02–1.37; *P* = 0.095) (Supplementary Table S2).

## Discussion

The primary finding of this study is that lower POD2ALB was associated with a higher incidence of pleural effusion in the donors from 2004 to 2012 when only synthetic colloid was administered during donor hepatectomy. Even after adjusting for PS matching, donors with POD2ALB ≤3.0 g/dL had considerably higher odds of developing postoperative pleural effusion than those with POD2ALB >3.0 g/dL. We also found that intraoperative use of albumin solution (from 2013 to 2014) resulted in significantly higher postoperative albumin levels and reduced incidence of postoperative pleural effusion than synthetic colloid use (from 2010 to 2012), before and after PS matching. Although the substitution of synthetic colloid with albumin solution was mainly due to the potential risk of AKI with the use of synthetic colloid, no association was found between the incidence of AKI and the type of colloid used.

Pleural effusion frequently occurs after liver resection in patients with various conditions^6,20,24,25^. In addition, postoperative albumin levels decrease in liver donors or patients with hepatocellular cancer (HCC) after hepatectomy^10,11^. Although hypoalbuminaemia is considered a contributing factor in the development of pleural effusion in various situations^17,18,21^, the direct impact of hypoalbuminaemia on pleural effusion has not been definitively identified because of the coexistence of additional risk factors for the development of pleural effusion^17,26^. Furthermore, despite the emphasis on the importance of donor safety, few reports have assessed the impact of early postoperative albumin level on the development of pleural effusion in previously healthy liver donors.

Recent studies have demonstrated that early postoperative hypoalbuminaemia is associated with postoperative complications^27,28^. This large study of 2316 liver donors, in agreement with findings from previous reports, revealed that early postoperative hypoalbuminaemia has an impact on postoperative complications such as pleural effusion, suggesting a close relationship between low albumin level and surgical stress or trauma that can predict adverse clinical outcomes after hepatectomy^27,28^.

Synthetic colloid was mainly used for volume replacement during donor hepatectomy from 2004 to 2012 at our institution. In that period, lower POD2ALB was associated with a higher incidence of postoperative pleural effusion in the liver donors. Our findings are in accordance with previous results showing the association between hypoalbuminaemia and pleural effusion in patients with HCC^24,25^. In addition, synthetic colloid was substituted by albumin solution during donor hepatectomy from 2013, due to the emerging evidence of adverse effects such as AKI with the use of synthetic colloids^22,23^. This replacement resulted in higher postoperative albumin levels and lower incidence of pleural effusion in LDLT donors from 2013 to 2014 compared to those from 2010 to 2012. In line with our findings, Kikuchi *et al*.^20^ reported that preoperative administration of branched-chain amino acids, which increase biosynthesis and secretion of albumin by hepatocytes, was effective in preventing ascites and pleural effusion, suggesting that albumin may have the effect of reducing oedema, ascites, and pleural effusion.

Contrary to the present study results, Schumann *et al*.^11^ reported that re-admissions for pleural effusion were not associated with albumin changes at the first and third weeks postoperatively in liver donors, although they admitted that the small sample size for their analysis might have prevented their results from reaching statistical significance. Tanaka *et al*.^29^ reported that preoperative albumin level was not associated with intractable pleural effusion after liver resection for HCC. Intractable pleural effusion was defined as the postoperative pleural effusion that developed within 1 month of surgery and that required thoracentesis. The results of our study may not be directly comparable with those from previous studies because of the differences in the time of albumin level measurements, definition of outcomes, or study populations.

Although the exact mechanism of pleural effusion after hepatectomy has not been fully elucidated, we presumed that the combination of hypoalbuminaemia and surgical techniques of liver resection might be linked to the development of postoperative pleural effusions. The level of albumin decreases mainly as a result of dilution secondary to fluid infusion and redistribution secondary to altered vascular permeability in the early perioperative period^13,14^. As albumin level decreases, its ability to sustain plasma COP and to scavenge free radicals (which impair the function of lymphatics in preventing oedema formation during inflammation) may decrease^12,30^. It has been suggested that in the absence of elevated hydrostatic pressure, an increase in capillary filtration owing to a decrease in plasma COP due to low albumin level may be compensated for by a greater increase in lymphatic flow^26,31^.

In the case of hepatectomy, postoperative portal hypertension occurs in patients with cirrhotic and noncirrhotic livers^32,33^, and relative portal hypertension may occur in liver donors^34,35^. In addition, surgical manipulation of the liver may cause significant damage to the lymphatic vessels^25,29,36^ and promote the formation of flow routes between the peritoneal and thoracic cavities^37^. In line with findings from previous reports^9,29^, our study also demonstrated that right hepatectomy, which entails extended division of ligaments containing lymphatic vessels and exposes a larger bare area, thus enhancing flow via the peritoneopleural communication, was associated with an increased incidence of pleural effusion. Notably, even among the donors undergoing right hepatectomy, POD2ALB ≤3.0 g/dL was associated with a higher likelihood of postoperative pleural effusion. Taken together, postoperative hypoalbuminaemia combined with the effect of surgical procedures of hepatic resection may have resulted in fluid accumulation in the peritoneal cavity and subphrenic collection, leading to pleural effusion in liver donors.

In this study, the chosen cutoff value of POD2ALB for PS matching was 3.0 g/dL. This cutoff is far above the value generally used to define hypoalbuminaemia as an aetiology of pleural effusion (e.g., serum albumin level ≤2.0 or ≤1.8 g/dL, respectively)^17,18^. In fact, early postoperative hypoalbuminaemia is common after various surgeries^21,27,28,38^, but pleural effusion occurs more frequently after hepatectomy than after other surgeries. Considering the combined effect of hypoalbuminaemia and surgical characteristics of hepatic resection on the development of pleural effusion as described above, clinically significant albumin levels may be higher than previously suggested as the cause of pleural effusion despite the need for further validation in multicentre studies. In addition, we found out that the prevalence of postoperative pleural effusion was lower in the donors infused with albumin solution, compared to those infused with synthetic colloid during donor hepatectomy. However, due to the retrospective design of the study, we could not confirm the causal relationship between albumin administration and reduction of the incidence of postoperative pleural effusion. Therefore, further studies are warranted to determine the effect of albumin solution on postoperative pleural effusion.

This study has some limitations. First, this was a retrospective observational study, and although we used the PS matching to control for selection bias, the effects of confounding factors may not have been entirely excluded. Second, because data were collected from a single centre, local intraoperative and postoperative management of liver donors may have influenced the clinical outcome. Therefore, further large-scale, multicentre studies are necessary to confirm the causality between hypoalbuminaemia and the development of pleural effusion after living donor hepatectomy.

In conclusion, this study suggests that early postoperative hypoalbuminaemia is associated with the development of pleural effusion in living donor hepatectomy. Further prospective trials are required to determine the effect of exogenous albumin administration on reducing the incidence of postoperative pleural effusion following living donor hepatectomy.

## Methods

This study was approved by the Institutional Review Board of Asan Medical Center [2015–1295], which waived the requirement for written informed consent due to the retrospective study design. Methods were performed in accordance with the relevant guidelines and regulations. The datasets generated during and/or analysed during the current study are available from the corresponding author on reasonable request.

### Donors

A total of 2353 living liver donors who underwent donor hepatectomy for LDLT at Asan Medical Center, Seoul, Republic of Korea, between January 2004 and January 2014, were enrolled. The Hospital Based Organ Procurement Organization designated by law procured living or deceased liver grafts, and none of the grafts were obtained from executed prisoners. We excluded donors who had previous pulmonary disease, renal disease, or incomplete data (Fig. 1).

### Anaesthetic technique

After the application of routine monitoring (electrocardiography, noninvasive blood pressure measurement, and pulse oximetry), anaesthesia was induced with intravenous thiopental, fentanyl, and vecuronium or rocuronium. After endotracheal intubation, mechanical ventilation was applied with a tidal volume of 8–10 mL/kg ideal body weight, inspiratory-to-expiratory ratio 1:2, and respiratory rate adjusted to maintain end-tidal partial pressure of carbon dioxide at 30–40 mmHg. Anaesthesia was maintained with volatile anaesthetics (sevoflurane or desflurane) in a mixture of 50% nitrous oxide and oxygen, along with intermittent boluses of fentanyl, and vecuronium or rocuronium.

Intraoperative fluid was maintained with crystalloid and colloid solutions (Voluven [Fresenius Kabi, Bad Homburg, Germany] or 20% human albumin [Green Cross Co., Yong-In, Korea]). Administration of fluid was limited during the dissection period at the surgeon’s discretion, and synthetic colloid was mainly used after liver resection for volume replacement until 2012. Considering the potential risk of AKI with the use of synthetic colloids^22,23^, Voluven was substituted by 20% albumin solution in LDLT donors at our institution from January 9, 2013. Throughout the surgery, systolic arterial blood pressure was maintained at >90 mmHg, urine output at >0.5 mL·kg^−1^·h^−1^, and haemoglobin concentration at >7.0 g/dL. For all donors, intravenous fentanyl patient-controlled analgesia was provided to control postoperative pain.

### Clinical data

Baseline characteristics, and laboratory, intraoperative, postoperative, and perioperative radiological data were acquired from the computerized databases of our institution (Electronic Medical Record System and Picture Archiving Communication System of Asan Medical Information System). Baseline characteristics included age, sex, BMI, and comorbidities (e.g., cardiovascular disease, pulmonary disease, and renal disease). Laboratory data included complete blood count; prothrombin time; and levels of creatinine, albumin, total bilirubin, aspartate aminotransferase, and alanine transaminase. Intraoperative data included type of surgical procedure (right or left hepatectomy), intraoperative fluids, volume of blood components, furosemide or ephedrine use, urine output, and duration of anaesthesia. The occurrence of postoperative pleural effusion was confirmed by comparing preoperative and postoperative chest radiographs taken daily from the day of surgery to POD 7.

### Definition of outcomes

The primary outcome of our study was the incidence of postoperative pleural effusion, confirmed through formal interpretation of chest radiographs by radiologists. Each of the chest PA radiographs was interpreted by two radiologists, and the presence of pleural effusion was determined based on a consensus between the two radiologists. In addition, the prevalence of AKI was assessed to determine its association with the type of colloid resuscitation. Postoperative AKI was defined using the KDIGO criteria for changes in serum creatinine within POD 7. According to the KDIGO criteria, AKI was defined as an increase in serum creatinine of ≥0.3 mg/dL within 48 hours or ≥1.5 times baseline, which is known or presumed to have occurred within the POD 7^39^. According to the LDLT protocol used at our institution, for donor safety, the routine postoperative hospital stay is 2 weeks, even if the donor is in good general condition. Therefore, we did not consider the length of hospital stay as a postoperative outcome.

### Statistical analysis

Variables are presented as mean (±standard deviation), median (interquartile range), or frequency (percentage). Intergroup differences were evaluated using Student’s t-test or Mann–Whitney U-test for continuous variables, and the chi-square or Fisher’s exact test for categorical variables, as appropriate.

Multiple logistic regression analysis that included the preoperative and intraoperative variables shown in Table 1 was used to identify the factors associated with postoperative pleural effusion. Variables with *P* < 0.1 in univariate analyses were entered into a stepwise-backward multivariate analysis. To reduce the impact of potential confounding factors in an observational study, we also performed rigorous adjustment for baseline differences by use of the PS matching^40,41^. The value of the 75th percentile of POD2ALB was used as a cut-off value. The PS was derived from a logistic regression model that included the perioperative variables shown in Table 1. Discrimination of the model was assessed by C statistics (0.829), and calibration was evaluated with the Hosmer-Lemeshow statistics (χ^2^ = 7.160; df = 8; *P* = 0.520). PS matching was performed by greedy matching with a caliper of 0.2 standard deviations of the logit of the PS. In the PS-matched cohort, we assessed the balance in baseline covariates between the two groups, using the paired t-test or Wilcoxon signed rank test for continuous variables, and McNemar’s test for categorical variables. The absolute standardized differences were used to diagnose the balance after PS. The risks of clinical outcome were assessed with logistic regression using generalized estimating equations that accounted for the clustering of matched pairs and weighted logistic regression.

Multiple logistic regression analysis of donors from 2010 to 2014 was performed to assess the effect of intraoperative use of 20% albumin solution instead of synthetic colloid on clinical outcomes. In addition, PS matching was performed to adjust the baseline differences between the groups classified by the type of colloid resuscitation. Model discrimination was assessed by C statistics (0.863), and calibration was evaluated with the Hosmer-Lemeshow statistics (χ^2^ = 5.786; *df* = 8; *P* = 0.671). A two-tailed *P*-value of <0.05 was considered significant. All statistical analyses were performed with SAS version 9.1 (SAS Institute Inc., Cary, NC, USA).

## Supplementary information

Supplementary Tables S1 and S2
