# Supplementary material for: Early postoperative hypoalbuminaemia is associated with pleural effusion after donor hepatectomy: A propensity score analysis of 2316 donors
Source: Sci Rep. 2019 Feb 26;9:2790. doi: 10.1038/s41598-019-39126-0 (PMC6391412; doi:10.1038/s41598-019-39126-0)
Supplement: Supplementary file 1 — Supplementary Tables S1 and S2 [file 41598_2019_39126_MOESM1_ESM.pdf]

Supplementary Materials

Early postoperative hypoalbuminemia is associated with pleural effusion after donor hepatectomy: A propensity score analysis of 2316 donors

Hye-Won Jeong, Jung-Won Kim, Won-Jung Shin, Seon-Ok Kim, Young-Jin Moon, Hye-Mee Kwon, Kyeo-Woon Jung, In-Gu Jun, Jun-Gol Song, Gyu-Sam Hwang

Contents

Supplementary Table S1. Baseline characteristics and intraoperative data of unmatched and propensity score matched donors from 2010 to 2014

Supplementary Table S2. Outcomes stratified by the use of albumin solution during donor hepatectomy

Supplementary Table S1. Baseline characteristics and intraoperative data of unmatched and propensity score matched donors from 2010 to 2014

	Unmatched (n = 986)			Propensity score matched (n = 386)			
Variables	Synthetic colloid (n = 730)	20% albumin solution (n = 256)	Standardized difference	Synthetic colloid (n = 193)	20% albumin solution (n = 193)	*P*-value	Standardized difference
Preoperative variables							
Age (years)	27.2 ± 7.6	28.5 ± 9.1	0.137	27.9 ± 7.8	27.6 ± 8.1	0.755	0.027
Sex, male	530 (72.6%)	171 (66.8%)	0.123	137 (71.0%)	132 (68.4%)	0.635	0.055
BMI (kg/m^2)	22.8 ± 2.8	23.0 ± 3.0	0.061	22.7 ± 2.9	22.8 ± 2.9	0.666	0.044
Haemoglobin (g/dL)	14.6 ± 1.5	14.6 ± 1.4	0.007	14.6 ± 1.5	14.6 ± 1.4	0.928	0.009
Platelet count (×10^9/L)	243.0 ± 48.5	240.3 ± 49.1	0.056	239.7 ± 53.4	241.2 ± 46.4	0.770	0.031
PT (INR)	1.00 ± 0.06	1.00 ± 0.06	0.022	1.00 ± 0.06	0.99 ± 0.06	0.602	0.051
Creatinine (mg/dL)	0.8 ± 0.2	0.8 ± 0.2	0.164	0.8 ± 0.2	0.8 ± 0.2	0.751	0.030
Albumin (g/dL)	4.4 ± 0.3	4.3 ± 0.4	0.277	4.4 ± 0.3	4.3 ± 0.3	0.836	0.018
Total bilirubin (mg/dL)	0.9 ± 0.4	0.7 ± 0.3	0.501	0.8 ± 0.3	0.7 ± 0.3	0.541	0.057
AST (IU/L)	19.5 ± 5.7	17.9 ± 4.8	0.335	18.0 ± 4.1	18.3 ± 4.9	0.554	0.059
ALT (IU/L)	17.5 ± 9.5	16.5 ± 9.3	0.110	16.5 ± 7.4	16.8 ± 9.9	0.833	0.029
Intraoperative variables							
Anaesthetic time (min)	450.1 ± 69.6	444.8 ± 75.2	0.070	446.8 ± 67.4	442.8 ± 75.4	0.573	0.053
Right hepatectomy	689 (94.4%)	213 (83.2%)	0.299	172 (89.1%)	170 (88.1%)	0.868	0.028
Crystalloid (L)	2942.1 ± 701.9	3131.8 ± 833.3	0.228	2983.4 ± 765.8	3032.5 ± 734.8	0.513	0.059
Synthetic colloid (mL)	569.7 ± 220.6	0.0 ± 0.0					
20% albumin solution (mL)	0.0 ± 0.0	200.9 ± 21.9					
Urine output (mL)	649.7 ± 323.2	741.3 ± 403.5	0.227	694.9 ± 353.1	724.3 ± 400.7	0.469	0.073
Diuretics use	166 (22.7%)	95 (37.1%)	0.297	69 (35.8%)	68 (35.2%)	1.000	0.011

Ephedrine use	200 (27.4%)	87 (34.0%)	0.139	65 (33.7%)	61 (31.6%)	0.749	0.044
Transfusion	1 (0.1%)	0 (0.0%)	0.052	0 (0.0%)	0 (0.0%)		

Values are expressed as mean ± standard deviation, or number of donors (%), as appropriate.

Abbreviations: BMI, body mass index; PT, prothrombin time; INR, international normalized ratio; AST, aspartate aminotransferase; ALT, alanine transaminase.

Supplementary Table S2. Outcomes stratified by the use of albumin solution during donor hepatectomy

	Type of colloids	Unadjusted				Multivariable adjusted[a]			Propensity score-matched			
		Event/n	OR	95% CI	*P*-value	OR	95% CI	*P*-value	Event/n	OR	95% CI	*P*-value
Pleural effusion	Albumin solution	83/256	0.63	0.46–0.84	0.002	0.55	0.39–0.77	<0.001	55/193	0.61	0.41–0.92	0.018
	Synthetic colloid	317/730	1			1			76/193	1		
Acute kidney injury	Albumin solution	1/256	0.16	0.02–1.17	0.070				1/193	0.16	0.02–1.37	0.095
	Synthetic colloid	18/730	1						6/193	1		

[a]Adjusted by all variables in Supplementary Table S1 except albumin solution and synthetic colloid.

Abbreviations: OR, odds ratio; CI, confidence interval.
